# Early prediction of the outbreak risk of dengue fever in Ba Ria-Vung Tau province, Vietnam: An analysis based on Google trends and statistical models

**DOI:** 10.1016/j.idm.2025.03.001

**Published:** 2025-03-05

**Authors:** Dang Anh Tuan, Pham Vu Nhat Uyen

**Affiliations:** a Faculty of Information Technology, Industrial University of Ho Chi Minh City, Ho Chi Minh City, 70000, Viet Nam; b Faculty of Chemical Engineering, Industrial University of Ho Chi Minh City, Ho Chi Minh City, 70000, Viet Nam

**Keywords:** Dengue fever, Google trends, Early forecasting, Outbreak, Ba Ria-Vung Tau, Statistical model, Machine learning, Big data

## Abstract

Dengue fever (DF), caused by the Dengue virus through the Aedes mosquito vector, is a dangerous infectious disease with the potential to become a global epidemic. Vietnam, particularly Ba Ria-Vung Tau (BRVT) province, is facing a high risk of DF. This study aims to determine the relationship between the search volume for DF on Google Trends and DF cases in BRVT province, thereby constructing a model to predict the early outbreak risk of DF locally. Using Poisson regression (adjusted by quasi-Poisson), considering the lagged effect of Google Trends Index (GTI) search volume on DF cases, and removing the autocorrelation (AC) of DF cases by using appropriate transformations, seven forecast models were surveyed based on the dataset of DF cases and GTI search volume weekly with the phrase "sốt xuất huyết" (dengue fever) in BRVT province from January 2019 to August 2023 (243 weeks). The model selected is the one with the lowest dispersion index. The results show that the correlation coefficient (95% confidence interval) and dispersion index of the 7 models including Basis TSR; Basis TSR + AC: Lag(Residuals,1); Basis TSR + AC: Lag(SXH,1); Basis TSR + AC: Lag(log(SXH+1),1); TSR Lag(GTI,2) + AC: Lag(log(SXH+1),2); TSR Lag(GTI,3) + AC: Lag(log(SXH+1),3); TSR Lag(GTI,0) + AC: Lag(log(SXH+1),1) are 0.71 (0.63–0.76) and 74.2; 0.79 (0.73–0.83) and 48.6; 0.89 (0.87–0.92) and 37.3; 0.98 (0.97–0.99) and 7.2; 0.96 (0.95–0.97) and 14.3; 0.93 (0.91–0.94) and 25.7; 0.98 (0.97–0.99) and 6.8, respectively. Therefore, the final model is the most suitable one selected. Testing the accuracy of the selected model using the ROC curve with the Youden criterion, the AUC (threshold 75%) is 0.982, and the AUC (threshold 95%) is 0.984, indicating the very good predictive ability of the model. In summary, the research results show the potential for applying this model in Vietnam, especially in BRVT, to enhance the effectiveness of epidemic prevention measures and protect public health.

## 1 Introduction

Dengue fever (DF) is an acute infectious disease with the potential for large-scale outbreaks. The disease is caused by the Dengue virus, transmitted through Aedes mosquitoes, primarily *Aedes aegypti* and *Aedes albopictus*. (WHO) Over the past two decades, DF incidence has risen significantly, with global cases increasing eightfold from 2000 to 2020 (Zeng et al., 2021). Vietnam, particularly Ba Ria-Vung Tau (BRVT) province, faces a high risk of DF outbreaks due to its tropical climate and urbanization (Tuan & Dang, 2024).

Traditional dengue surveillance systems rely on clinical case reporting, which often suffers from delayed data collection and reporting inefficiencies (Runge-Ranzinger et al., 2014). To address this limitation, recent studies have explored the potential of big data sources such as Google Trends Index (GTI) for real-time disease monitoring (Sylvestre et al., 2022). However, prior research primarily focused on correlation-based analyses, lacking robust predictive modeling frameworks that integrate multiple influencing factors (Gluskin et al., 2014). Additionally, these studies often fail to account for regional variations in internet penetration and search behaviors, limiting the generalizability of their findings (Gluskin et al., 2014).

This study aims to bridge these research gaps by introducing a comprehensive GTI-based forecasting model that improves upon existing methodologies. The key innovations of this study include.

• Advanced GTI data processing techniques that mitigate autocorrelation and enhance predictive stability.
• Optimization of lag periods through rigorous cross-correlation analysis to identify the most predictive timeframes.
• Integration of a dispersion index minimization approach to refine model accuracy and robustness.
• Performance benchmarking against traditional surveillance models to assess predictive improvements over conventional methods.

By addressing these limitations, this study presents a significant advancement in dengue forecasting, offering a more reliable and scalable model that can be adapted to other regions with similar epidemiological profiles. The findings contribute to enhancing early warning systems, allowing for timely public health interventions and better resource allocation during dengue outbreaks.

## 2 Material and methods

### 2.1 Study design

The study is designed based on correlation analysis, conducted in BRVT province from January 2019 to August 2023. The study population includes both target and sample populations. The target population is determined based on data on DF cases and Internet search queries with the phrase "sốt xuất huyết" (dengue fever) as reported by Google Trends, in the BRVT area. The sample population consists of data on DF and search volumes from 2019 to 2023. The sample size is calculated based on weekly data during the study period. The sampling technique applied is a complete enumeration, with specific criteria for sample selection and type defined. To control for selection bias, DF data are defined by hospital discharge diagnoses and provided by the Center for Disease Control (CDC) of BRVT province. Google Trends Index (GTI) data are accurately collected from Google Trends, with data entry controlled to minimize errors.

### 2.2 Data processing

**Fig. 1:**
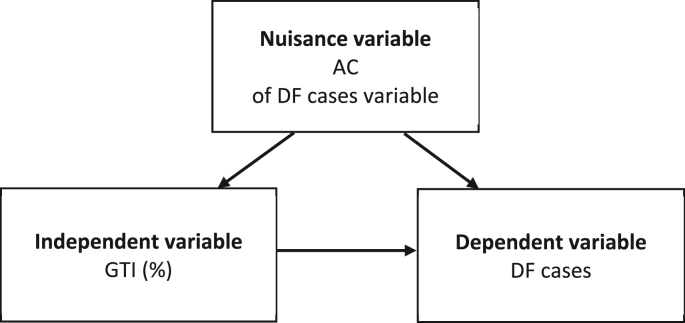
Relationship between variables in data processing.

Variables are listed and specifically defined. The independent variable is GTI (%), a quantitative variable representing the search query volume on the Internet with the phrase "sốt xuất huyết" (dengue fever). GTI data are collected from Google Trends, focusing on the geographical area of BRVT province during the study period. This variable ranges from 0 to 100%, where the highest value of 100% indicates the week with the highest search query volume, while a value of 0% represents a week with no search data. The dependent variable is DF cases, a quantitative variable representing the total number of diagnosed DF cases in a week. Data on DF cases are maintained in the surveillance system of the CDC of BRVT province. The nuisance variable is autocorrelation (AC), which is the frequent correlation between observations in a time series. In this study, the lag amplitude of DF cases is used to control this AC, ensuring independence between observations. (Fig. 1).

The data processing method involves importing and processing data using Excel 2016, with missing weeks calculated by averaging data from adjacent weeks. A year is accurately defined as consisting of 52 weeks, with the 53rd week excluded to ensure data consistency. Finally, the data are managed and analyzed using R 4.2.3 software.

### 2.3 Data analysis

#### 2.3.1 Descriptive statistics

We report the mean, standard deviation, minimum and maximum values, and the 25%, 50%, and 75% quartiles ranges of quantitative variables, including DF cases and GTI search volume on the Internet. Additionally, we describe the variation of variables over time through graphs and examine the correlation between them using Pearson or Spearman correlation coefficients depending on the distribution of the data.

#### 2.3.2 Analytical statistics

We conducted two main stages. In the first stage, we identified the relationship between GTI search volume and DF cases using Poisson regression, adjusted by quasi-Poisson. In this model, we considered the lagged effect of GTI search volume on DF cases and eliminated the AC of DF cases using appropriate transformations. The general model is represented as follows:

$$Y_{t∼}\;quasi-poisson\;\left(\mu_{t}\right)$$

$$\log\;\mu_{t}=\alpha +{\beta_{1}\;Lag\;GTI}_{t-k}{+\beta }_{AC}AC$$

$${=\text{Basis}\;\text{TSR}+\beta }_{AC}AC$$

Where.

• Y_t_: predicted DF cases in week t
• μ_t_: average of DF cases predicted by the Poisson model
• ${Lag\;GTI}_{t-k}$: GTI search volume in week t with k-weeks lag time (k = 0, 1, 2, 3)
• ${\;\alpha ,\beta }_{1},\beta_{AC}$: regression coefficient
• Basis TSR (Time Series Regression): baseline forecasting model
• AC (auto-correlation): the autocorrelation of the DF cases variable, respectively, consists of the residuals of Y_t-1_, Y_t_, and log(Y_t-1_+1)

In the second stage, we constructed a forecasting model for DF cases using Poisson regression, adjusted by quasi-Poisson, to identify the relationship between GTI search volume and DF cases, aiming to remove the autocorrelation of the DF cases variable. We investigated a total of seven forecasting models, with the model having the lowest dispersion index being selected as it provided the best forecasting results (Imai et al., 2015). During this process, we also transformed the selected model into a scoring scale by categorizing the GTI search volume predictor into corresponding quartile groups (<50, 50 – < 75, 75 – < 95, and ≥95) and examined the forecasting model based on these groups. The estimated coefficient β, computed from the standardized and rounded model, served as the forecasting score. Additionally, we considered a similar study to determine the cutoff point for classifying weeks as epidemic or non-epidemic based on DF cases. Accordingly, a week was considered epidemic when DF cases was 95% or more compared to the actual number (Phung et al., 2015). After constructing the model, we evaluated its predictive ability using the Receiver Operating Characteristic (ROC) curve. We used the Youden index to determine the optimal cutoff point on the ROC curve, where the model performed best, as well as calculated the percentage of accuracy predictions at this cutoff point. This helped us assess the performance of the DF case forecasting model and determine its suitability for predicting epidemic situations. The seven models implemented in this study include.

1. **Basis TSR**: The linear relationship between DF cases and 1-week lag time of GTI search volume (baseline model).
2. **Basis TSR + AC: Lag(Residuals,1)**: The linear relationship between DF cases and 1-week lag time of GTI search volume, removing the autocorrelation of DF cases by 1-week lag time of the resudials of the baseline model.
3. **Basis TSR + AC: Lag(SXH,1)**: The linear relationship between DF cases and 1-week lag time of GTI search volume, removing the autocorrelation of DF cases by 1-week lag time of DF cases.
4. **Basis TSR + AC: Lag(log(SXH+1),1)**: The linear relationship between DF cases and 1-week lag time of GTI search volume, removing the autocorrelation of DF cases by 1-week lag time of the logarithm of DF cases add 1 (adding 1 to DF cases to remove data with a value of 0)
5. **TSR Lag(GTI,2) + AC: Lag(log(SXH+1),2)**: The linear relationship between DF cases and 2-weeks lag time of GTI search volume, removing the autocorrelation of DF cases by 2-weeks lag time of the logarithm of DF cases add 1
6. **TSR Lag(GTI,3) + AC: Lag(log(SXH+1),3)**: The linear relationship between DF cases and 3-weeks lag time of GTI search volume, removing the autocorrelation of DF cases by 3-weeks lag time of the logarithm of DF cases add 1
7. **TSR Lag(GTI,0) + AC: Lag(log(SXH+1),1)**: The linear relationship between DF cases and GTI search volume, removing the autocorrelation of DF cases by 1-week lag time of the logarithm of DF cases add 1

Finally, we controlled for noise by examining the autocorrelation of variables and implementing mathematical transformations in the model to minimize this phenomenon. We relied on dispersion, sensitivity, and specificity to assess the strength of the model.

## 3 Result

### 3.1 Descriptive statistics

During the period from January 2019 to August 2023, a total of 34,658 cases of DF were reported in BRVT province. Both DF cases and GTI variables exhibit relatively wide dispersion, with the mean exceeding the median, indicating a Poisson distribution of the data. The minimum and maximum of DF cases in a week were 2 and 895 cases, respectively. The mean of DF cases per month was 143 cases. The mean weekly GTI search volume was 11.3 searches, with the minimum and maximum volumes being 0 and 100 searches, respectively (Table 1).

**Table 1:** Characteristics of DF cases and GTI search volume in BRVT province during the period from January 2019 to August 2023.

Variable	Mean±SD	Min	Quartile range (%)			Max
			25	50	75	
SXHD	143±218	2	23.5	44	103.5	895
GTI	11.3±17.3	0	0	5	11	100

**Fig. 2:**
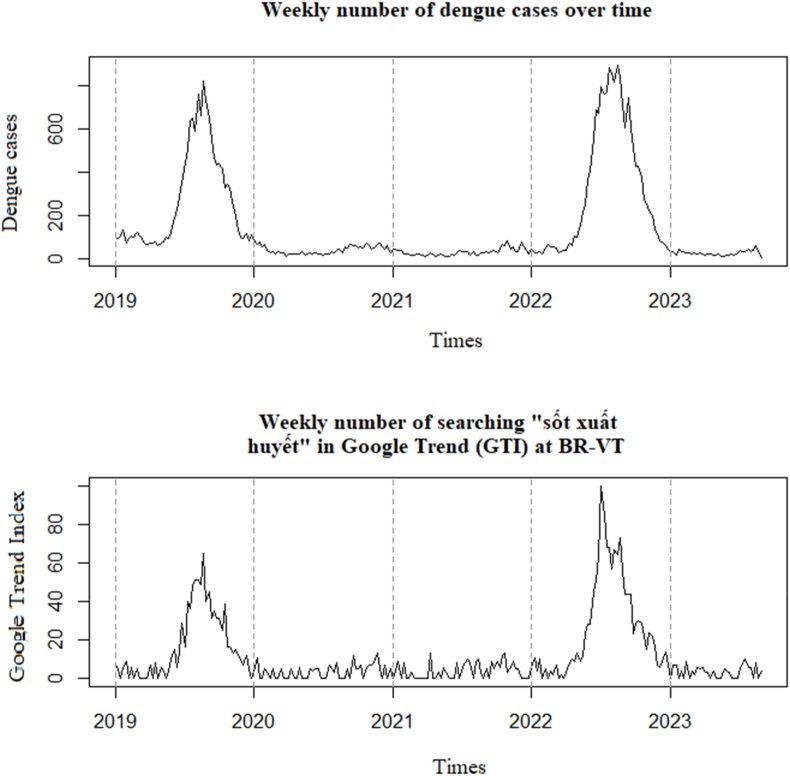
Distribution of DF cases and GTI search volume over time in BRVT province from January 2019 to August 2023.

The analysis of the distribution of DF cases and GTI search volume over time from 2019 to 2023 (Fig. 2) reveals an increasing trend in both DHF cases and GTI search volume. These two curves exhibit a similar upward trend, indicating a correlation between DF cases and GTI search volume. This correlation can be attributed to the increased awareness of the DF outbreak among the population, leading them to search for information about the disease more frequently. Additionally, the surge in DF cases may also prompt people to become more concerned and seek information about the disease more actively.

Additionally, Fig. 2 also indicates that DF cases tends to peak in the middle of the year and extends towards the end of the year, showing no clear cyclical pattern for the years 2019 and 2022 - which were marked by major outbreaks. GTI search volume experiences a slight increase in the middle of the year, extending towards the end of the year during the period from January 2019 to August 2023, with notable spikes also observed in 2019 and 2022.

### 3.2 Correlation between variables

**Fig. 3:**
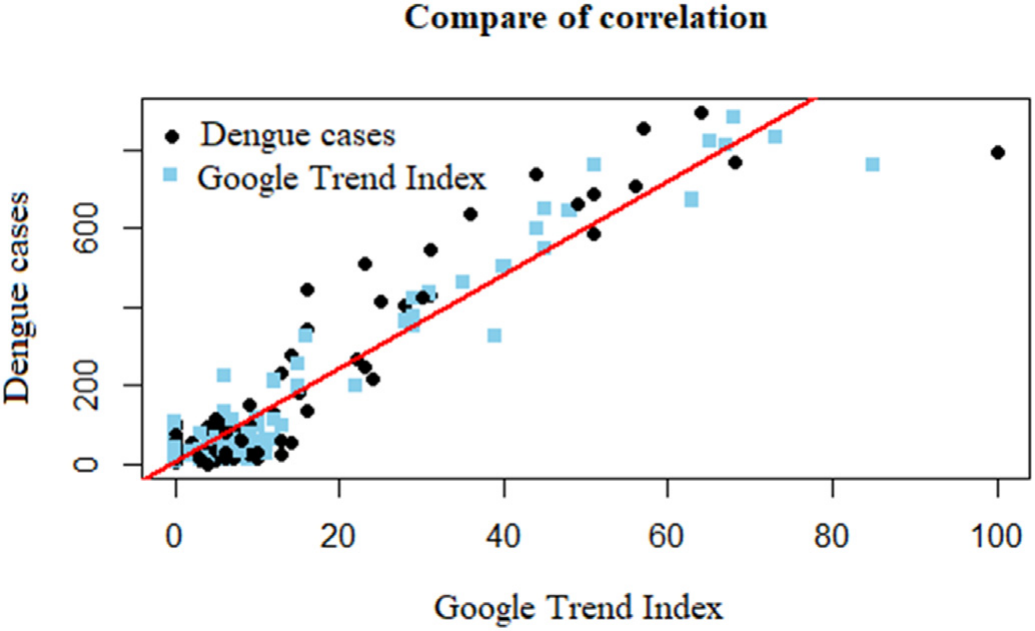
Scatter plot depicting the correlation between DF cases and the GTI search volume.

The correlation analysis between variables (Fig. 3) reveals that the linear regression line is a straight line passing through the average of the data points. The correlation coefficient is 0.94 (p < 0.001) with a 95% confidence interval of 0.93–0.96, indicating a strong positive correlation between DF cases and the GTI search volume.

**Fig. 4:**
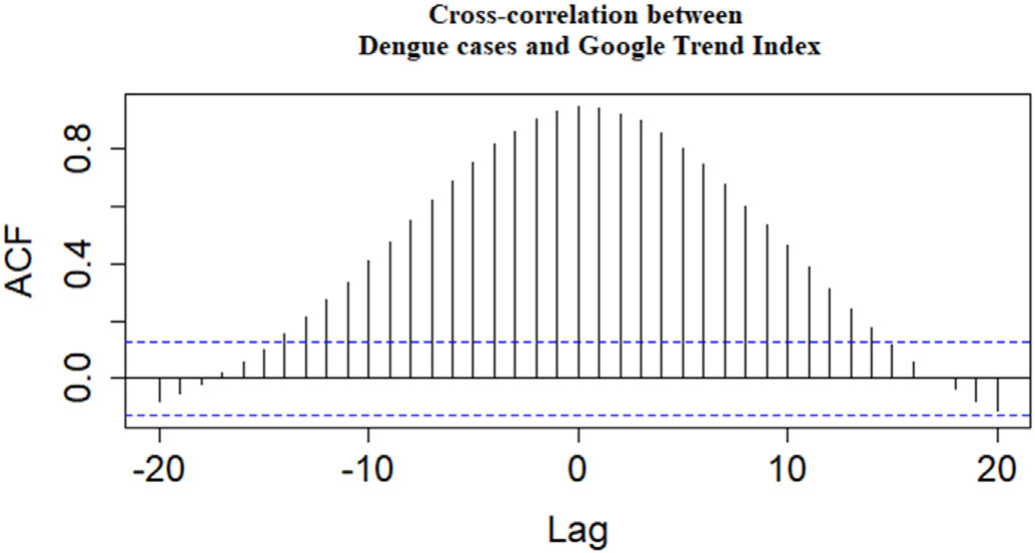
Cross-Correlation between DF cases and GTI.

To assess the level of correlation in the data and evaluate the lag time between the comparison values, we constructed a cross-correlation function to measure the degree of correlation between the variables over time (Fig. 4). Regarding the correlation level, the autocorrelation function (ACF) of DF cases tends to decrease gradually with lag time, indicating a decreasing correlation between DF cases values over time. The ACF of GTI oscillates around 0, suggesting no clear correlation between GTI values over time. However, we observe that the ACF of DF cases is higher than that of GTI, indicating a higher correlation between DF cases values compared to GTI. The ACF of DF cases tends to decrease with lag time, while the ACF of GTI oscillates around 0. Regarding lag time, the ACF of DF cases reaches its highest value at lag time 2, indicating that DF cases are most strongly correlated with cases occurring two weeks prior. The ACF of GTI reaches its highest value at lag time 1, indicating that GTI is most strongly correlated with DF cases occurring one week prior. Thus, the optimal lag time for correlation with DF cases is 2 weeks, while the optimal lag time for correlation between GTI and DF cases is 1 week. Therefore, lag times of 0, 1, 2, and 3 weeks for GTI represent lag times with the strongest correlation with DF cases.

**Fig. 5:**
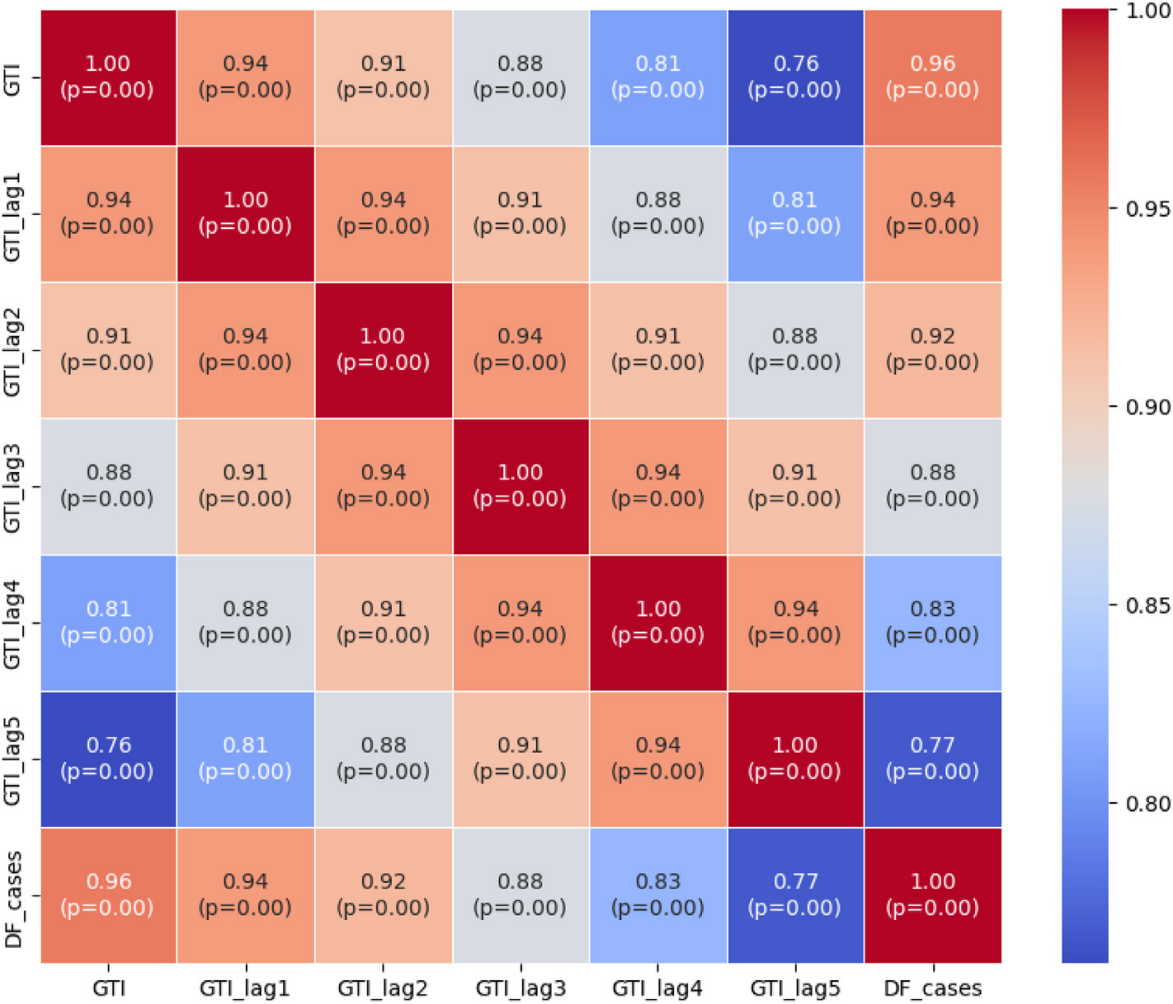
Correlation heatmap + p-value between GTI and dengue cases.

To further illustrate these findings, we have incorporated a heatmap visualization (Fig. 5) that highlights the correlation strength across various lag periods. This heatmap provides a comprehensive overview of the temporal relationship between GTI data and reported DF cases, aiding in the selection of the most effective forecasting timeframes.

These results validate the importance of lag period optimization in GTI-based disease prediction and enhance the interpretability of our model.

### 3.3 Relationship between DF cases and GTI

Model 1 Basis TSR

**Fig. 6:**
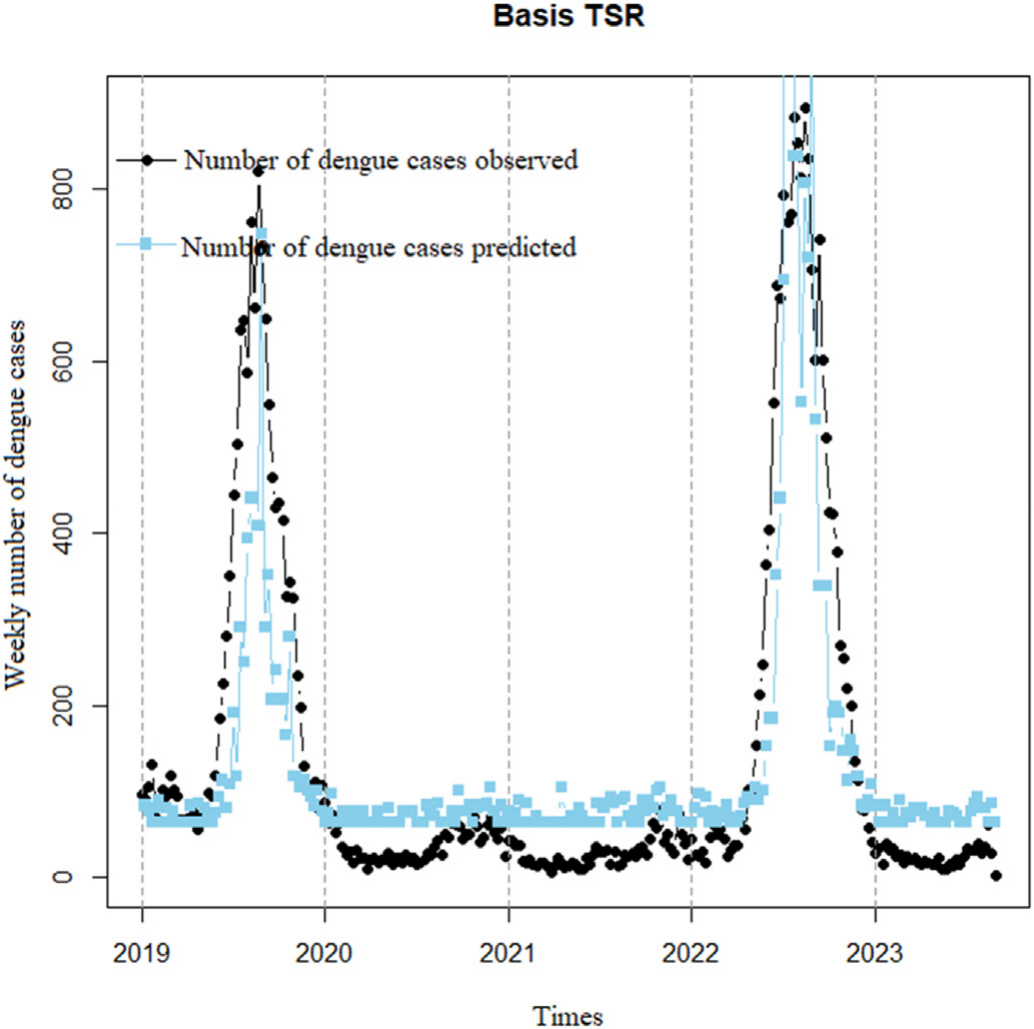
Linear regression time series foundational model.

**Fig. 7:**
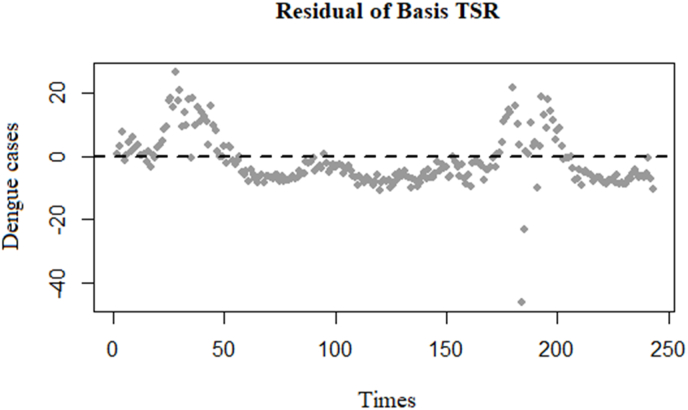
Residuals of the Basis TSR model.

Built using a Poisson model based on the linear relationship between DF cases and 1-week lag time of GTI (Fig. 6). From the chart, we observe a certain degree of similarity between DF cases and GTI search volume, with both curves showing an upward trend from 2019 to 2023. This suggests that an increase in DF cases often accompanies an increase in GTI search volume. Additionally, the chart also indicates that the variability of DF cases is higher than that of GTI search volume; there are some instances where DF cases rise significantly while GTI search volume does not increase correspondingly. With a correlation coefficient of 0.71 (p < 0.001) and a 95% confidence interval of 0.63–0.76, the model's dispersion is very high (74.2), and the predicted DF cases do not capture the actual data well, as evidenced by the residual plot (Fig. 7).

The residuals of the Basis TSR model exhibit non-random distribution around the zero line (Fig. 7), indicating a clear cyclic trend as time progresses. Consequently, it can be inferred that the Basis TSR model demonstrates a strong autocorrelation of the DF cases variable.

Model 2 Basis TSR using a 1-week lag time of residuals to remove autocorrelation

**Fig. 8:**
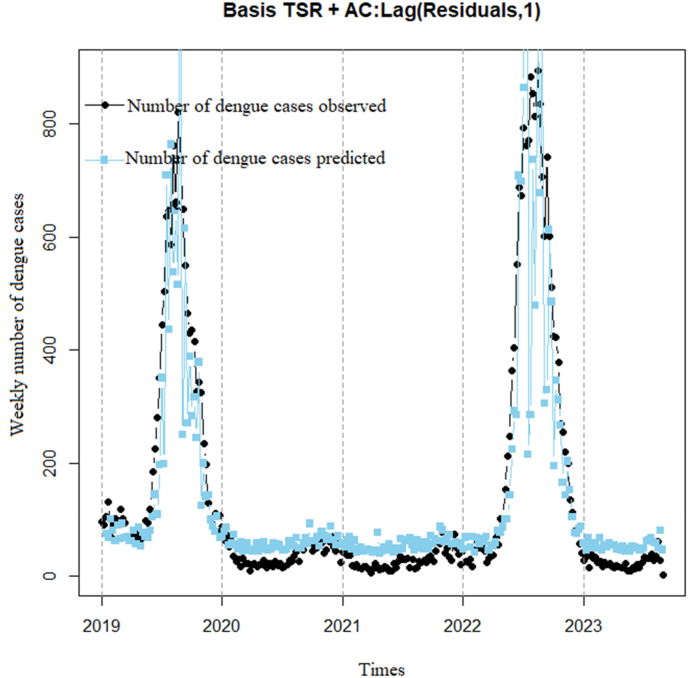
Basis TSR using a 1-week lag time of residuals.

Basis TSR model is recalculated after being augmented by a 1-week lag time of residuals from the Basis TSR model to adjust estimates aiming to remove autocorrelation and improve the capture of forecast values (recursive estimation method) (Fig. 8). The model shows that the observed DF cases tend to increase from 2019 to 2023; the predicted DF cases show a similar trend to the observed cases, although there are some differences, especially in 2022 and 2023. The correlation coefficient is 0.79 (p < 0.001) with a 95% confidence interval of 0.73–0.83. The dispersion index has relatively decreased but remains very high (48.6). Removing the autocorrelation of DF cases by using residuals can be iteratively performed until the estimated values approximate a graph closely resembling the actual DF cases, potentially increasing efficiency (i.e., reducing variance).

Model 3 Basis TSR using a 1-week lag time of DF cases to remove autocorrelation

**Fig. 9:**
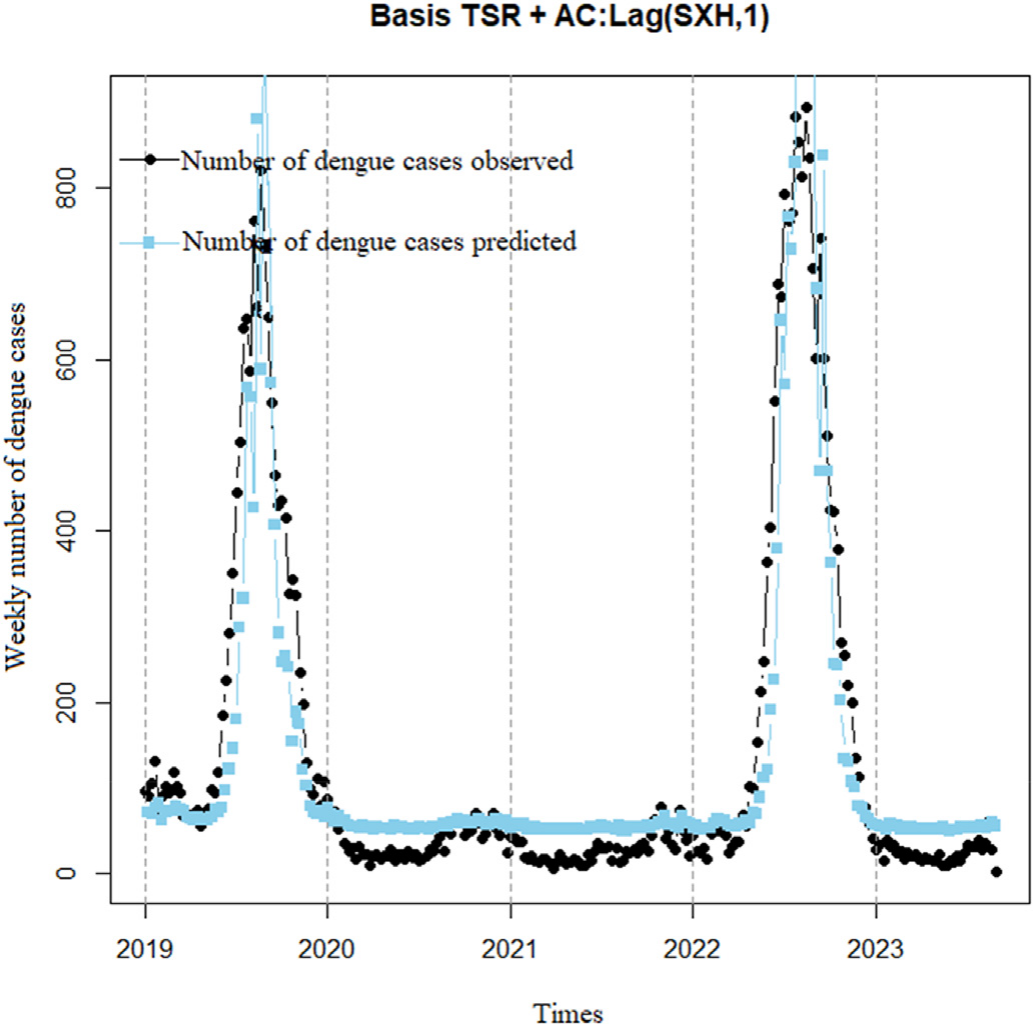
Basis TSR model with an added 1-week lag time of DF cases.

Basis TSR model after being augmented with a 1-week time lag of DF cases to remove autocorrelation between the two variables (Fig. 9). The graph illustrates that observed DF cases (black line) show a gradual increase from 2019 to 2023, while predicted DF cases (blue line) also demonstrate a rising trend over the same period. However, the predicted DF cases tend to be lower than the observed DF cases in most weeks. Although the correlation coefficient is 0.89 (p < 0.001) with a 95% confidence interval of 0.87–0.92, indicating relatively good correlation, the dispersion index remains relatively high (37.3), indicating that the predicted line does not capture the observed DF cases well.

Model 4 Basis TSR model utilizing a 1-week lag of the logarithm of DF cases add 1 to remove autocorrelation.

**Fig. 10:**
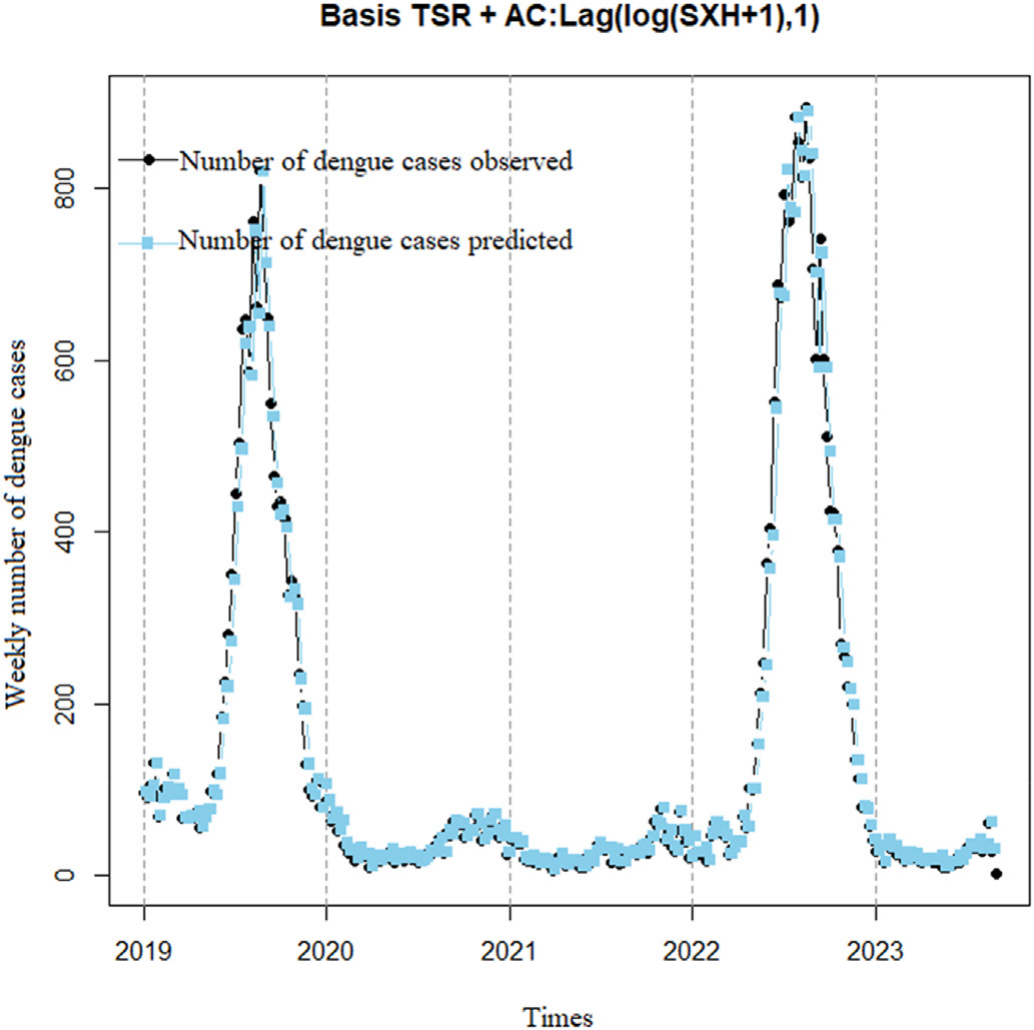
Basis TSR incorporating a 1-week lag time of the logarithm of DF cases add 1.

Basis TSR model incorporating a 1-week lag time of the logarithm of DF cases add 1 to mitigate the impact of zero values on the logarithmic transformation. The addition of 1 aims to alleviate issues arising from zero values when applying the logarithmic transformation. The lag time of 1 week was chosen to account for the delayed effect of DF cases on the search volume (Fig. 10). The plot illustrates that observed DF cases (black line) increase gradually from 2019 to 2023, peaking in 2022 with the highest number of cases across the years, experiencing a slight decrease in 2023 and showing a rising trend again from early 2023 onwards. Predicted DF cases (blue line) provide relatively accurate forecasts for most weeks compared to observed cases. The model exhibits a highly significant correlation coefficient of 0.98 (p < 0.001) with a 95% confidence interval of 0.97–0.99, indicating that the predicted line captures DF cases well, with a low dispersion index (7.2).

Model 5 TSR model correlating DF cases with a 2 weeks lag time of GTI, utilizing a 2 weeks lag time of the logarithm of DF cases add 1 to remove autocorrelation

**Fig. 11:**
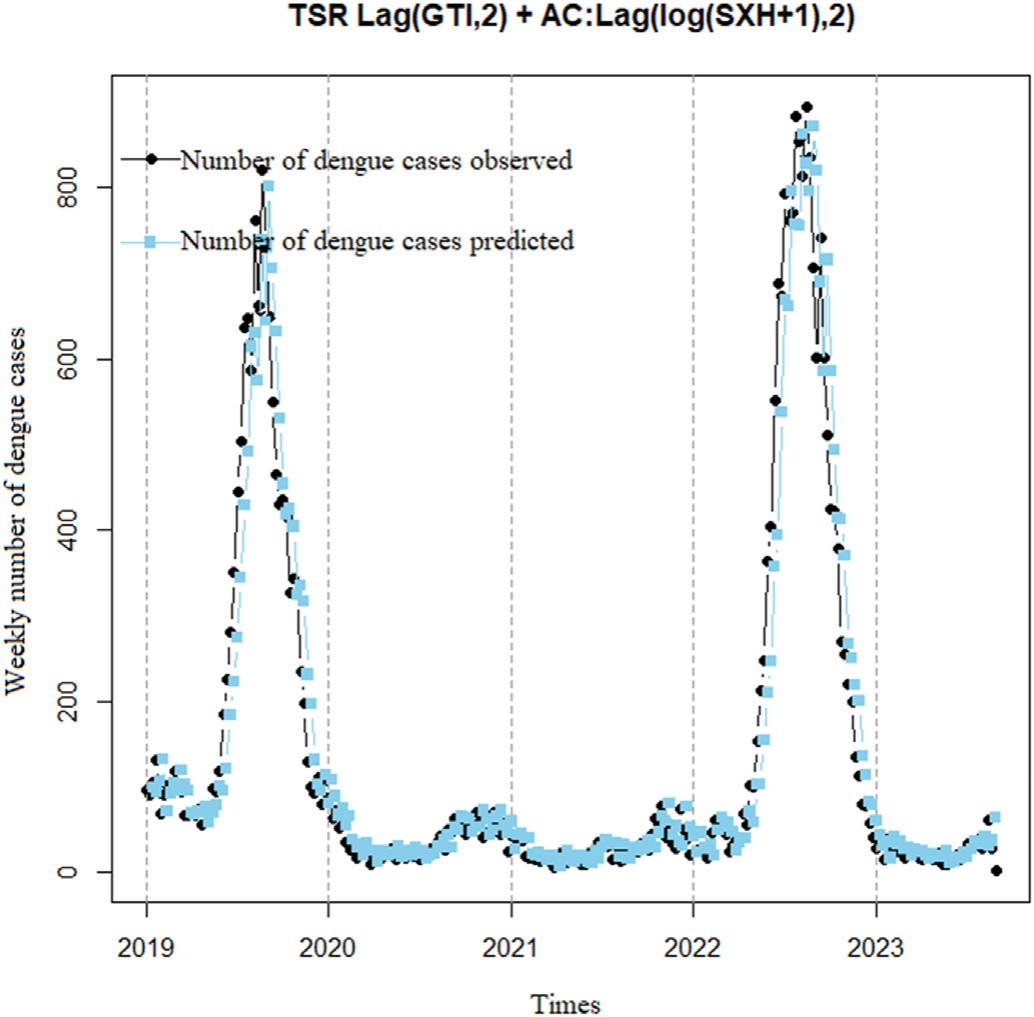
TSR model correlation between DF cases and 2 weeks lag time of GTI using 2 weeks lag time of the logarithm of DF cases add 1 to remove autocorrelation.

The graph (Fig. 11) illustrates a rising trend in DF cases from 2019 to 2023. Predicted DF cases also show an increasing trend but are lower than observed cases. The graph also reveals a pattern of increasing DF cases during the rainy season (from May to October) and decreasing during the dry season (from November to April). Moreover, the model analysis yields a correlation coefficient of 0.96 (p < 0.001) with a 95% confidence interval of 0.95–0.97, indicating that the predicted line adequately captures the observed cases. However, the dispersion index is higher (14.3) compared to Model 4.

Model 6 TSR model correlation between DF cases and 3 weeks lag time of GTI using 3 weeks lag time of the logarithm of DF cases add 1 to remove autocorrelation

**Fig. 12:**
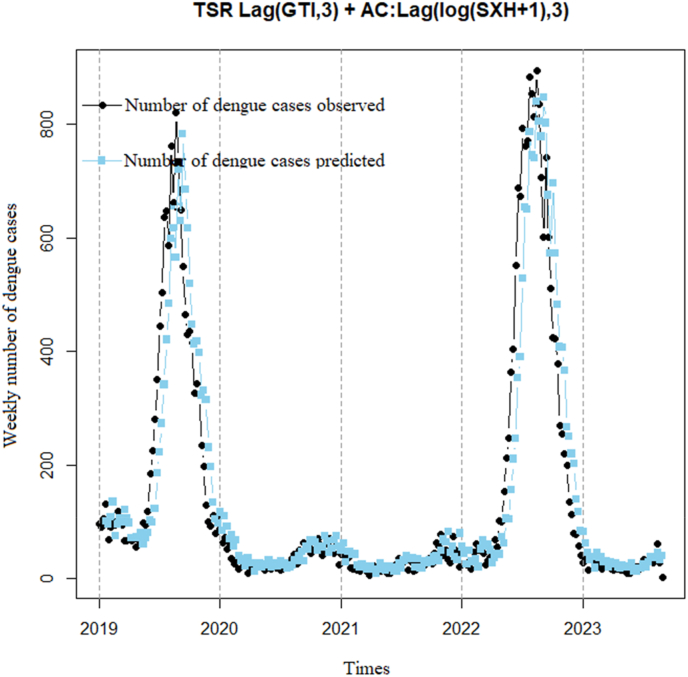
TSR model correlation between DF cases and 3 weeks lag time of GTI using 3 weeks lag time of the logarithm of DF cases add 1 to remove autocorrelation.

Fig. 12 shows that the observed cases tend to increase from 2019 to 2020, then decrease in 2021 and 2022. The predicted cases also show a similar trend, but with varying degrees of fluctuation. The model analysis yields a correlation coefficient of 0.93 (p < 0.001) with a 95% confidence interval of 0.91–0.94, indicating that the model can accurately predict the general trend of dengue cases but not the specific fluctuations in actual cases, as evidenced by the dispersion index not showing significant improvement (25.7).

Model 7 TSR model correlation between DF cases and 0 week lag time of GTI using 1 week lag time of the logarithm of DF cases add 1 to remove autocorrelation

**Fig. 13:**
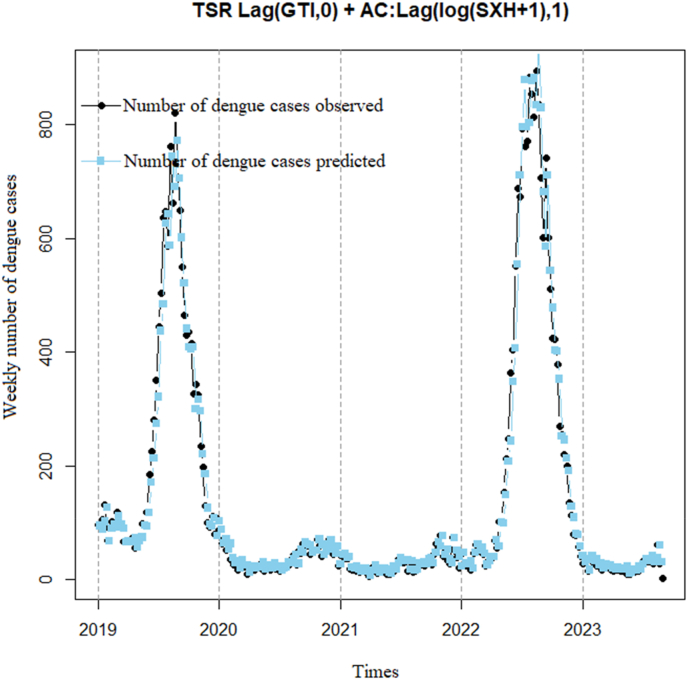
TSR model correlation between DF cases and 0 week lag time of GTI using 1 week lag time of the logarithm of DF cases add 1 to remove autocorrelation.

Fig. 13 illustrates a steady increase in DF cases over time. In 2019, reported DF cases fluctuated between 200 and 400 cases per week. In 2020, there was a significant increase in DF cases, reaching over 800 cases per week at its peak. This trend continued into 2021 and 2022. The predictive model suggests that this upward trend may persist in the future. The model predicts that DF cases will peak in 2023, followed by a gradual decrease. Model analysis yields a correlation coefficient of 0.98 (p < 0.001) with a 95% confidence interval of 0.97–0.99, indicating that the model captures actual cases very effectively, with a significantly improved dispersion index (only 6.8).

### 3.4 Table statistics of the analysis indices for the 7 linear Poisson models correlating dengue cases and GTI search volume

From Table 2, we observe that Model 7 is the most suitable model and should be chosen because it has the lowest dispersion index (only 6.8). A low dispersion index leads to better capability to capture the actual cases (Fig. 13).

**Table 2:** Table statistics of the analysis indices for the 7 linear Poisson models correlating dengue cases and GTI search volume.

No.	Model	r	StdE	p-value	Dispersion
1	Basis TSR	0.038	0.001	<0.001	74.2
2	Basis TSR + AC: Lag(Residuals,1)	0.036	0.001	<0.001	48.6
3	Basis TSR + AC: Lag(SXH,1)	−0.005	0.003	0.115	37.3
4	Basis TSR + AC: Lag(log(SXH+1),1)	0.001	0.001	0.292	7.2
5	TSR Lag(GTI,2) + AC: Lag(log(SXH+1),2)	0.001	0.001	0.61	14.3
6	TSR Lag(GTI,3) + AC: Lag(log(SXH+1),3)	0.001	0.002	0.585	25.7
**7**	**TSR Lag(GTI,0) + AC: Lag(log(SXH+1),1)**	**0.001**	**0.001**	**<0.001**	**6.8**

### 3.5 Transformation of the selected model into score points

After selecting the appropriate model (Model 7), we proceeded to transform the selected model into score points (Table 3). The results show that the predictor variable, GTI search volume, was divided into corresponding groups based on quartile ranges: <50, 50 – < 75, 75 – < 95, and ≥95. Subsequently, the selected forecasting model was represented through these classified groups. The estimated coefficient β was calculated from the standardized model and rounded to form the prediction points.

**Table 3:** Model prediction score points.

Grouping	Correlation coefficient	95% CI	Points
**GTI 0 week lag time (%)**			
<5	0		
5-11	0.09	0.88–1.35	9
11-51	0.94	2.06–3.22	94
≥51	1.39	3.07–5.19	139
**DF cases 1 week lag time (take the logarithm)**			
<3.8	0		
3.8–4.7	0.84	1.88–2.87	84
4.7–6.6	1.82	4.85–7.91	182
≥6.6	2.17	6.64–11.60	217

### 3.6 Testing the accuracy of the prediction model

**Fig. 14:**
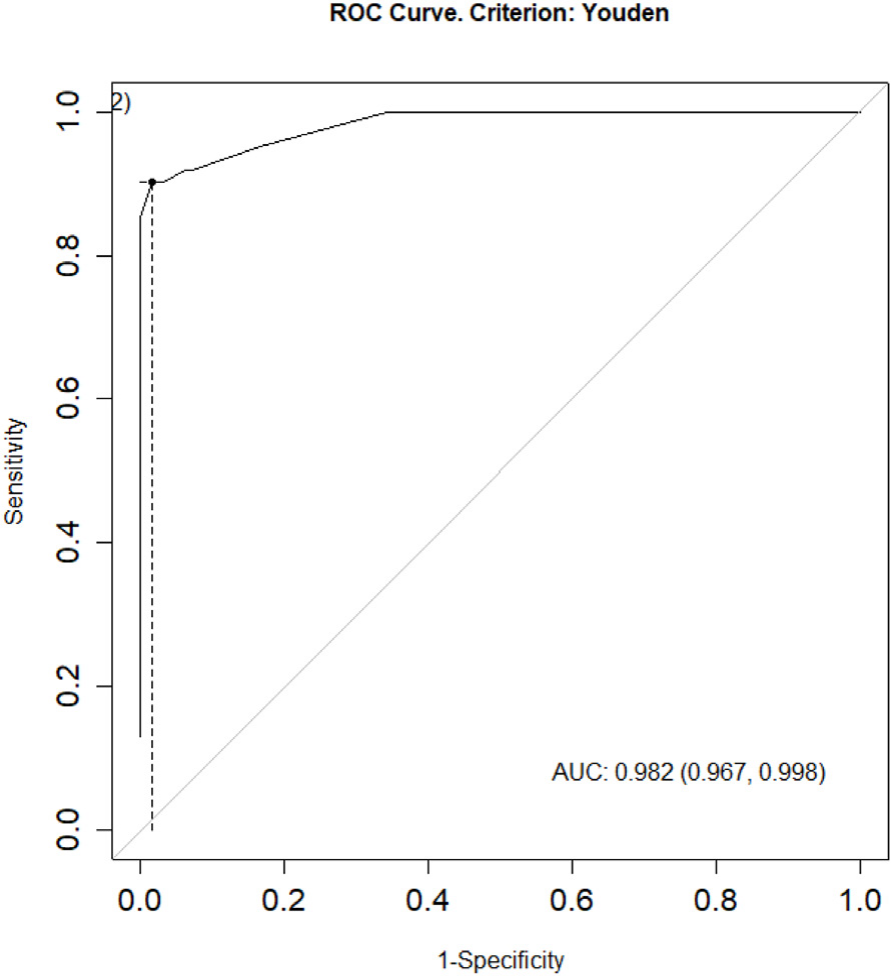
ROC curve at the 75% epidemic threshold.

**Fig. 15:**
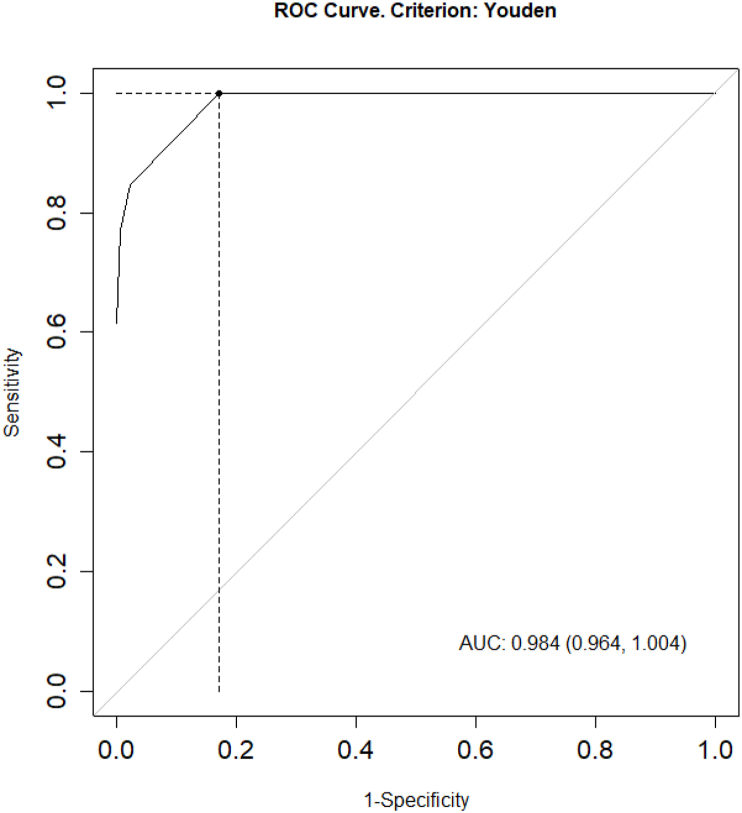
ROC curve at the 95% epidemic threshold.

To assess the accuracy of the prediction model, we conducted tests at two epidemic thresholds (75% and 95%). For the 75% threshold, the study divided the DF cases into two groups: there was an epidemic when the DF cases fell within the range of ≥75% of the actual cases (≥103.5 cases), and the remaining group was considered non-epidemic. The result of the area under the curve (AUC) was obtained as 0.982 (Fig. 14), indicating a very good predictive ability of the model. For the 95% threshold, the study also divided the DF cases into two groups: there was an epidemic when the DF cases fell within the range of ≥95% of the actual cases (≥705 cases), and the remaining group was considered non-epidemic. The result of the area under the curve (AUC) was obtained as 0.984 (Fig. 15), indicating a very good predictive ability of the model.

## 4 Discussion

### 4.1 The correlation between the DF cases and GTI search volume

The study results indicate a correlation between the DF cases and GTI search volume for the term "sốt xuất huyết" (dengue fever) with a correlation coefficient of 0.94 (95% confidence interval 0.93–0.96). This finding is consistent with previous studies by Atina Husnayain, Gunther Eysenbach, Daniel Romero-Alvarez, Emmanuelle Sylvestre, Kangkang Liu, and Zhihao Li, as well as Tran Ngoc Dang (Husnayain et al., 2019, 2020; Li et al., 2017; Liu et al., 2016; Romero-Alvarez et al., 2020; Syamsuddin et al., 2020a; Sylvestre et al., 2022; Đăng, 2019). Among these studies, those conducted in tropical regions have shown correlation values ranging from 0.68 to 0.95 (Husnayain et al., 2019, 2020; Liu et al., 2016; Đăng, 2019). The increased demand for information during disease outbreaks, including preventive measures, symptoms, and the disease situation, could be a contributing factor.

However, the results of this study, showing a high correlation between DF cases and GTI search volume, differ somewhat from previous research by Gianfranco Cervellin and Nicola Luigi Bragazzi (Alicino et al., 2015; Cervellin et al., 2017). These studies not only found differences between Google Trends data and disease incidence but also highlighted the significant influence of media coverage on Google Trends data rather than the actual disease burden. In other words, Google Trends data is influenced by media coverage, internet penetration, and mobile phone usage frequency. Additionally, a finding in Chan's study indicated that information-seeking behavior related to DF is less affected by media coverage (Chan et al., 2011).

### 4.2 Dengue prediction model based on Google trends

In total, seven different models were constructed based on the linear relationship between the actual DF cases and the lag times of GTI search volumes, along with complex transformations to remove the autocorrelation between DF cases over time. The effectiveness of each model was classified and selected based on the dispersion index, a metric commonly used to evaluate the forecasting ability of a model (Imai et al., 2015). This implies that a model with a lower dispersion value corresponds to better forecasting ability. With a dispersion value of 6.8, the linear regression model between DF cases and GTI search volume within the same week, removing the autocorrelation of DF cases by the 1-week lag time of logarithm of DF cases add 1 (TSR Lag(GTI,0) + AC: Lag(log(SXH+1),1)), was identified as the best forecasting model for DF cases in BR-VT province. This means that the best model of the study could be practically applied to forecast DF outbreaks occurring within the same week, with a high correlation coefficient of 0.98 (p < 0.001) and a 95% confidence interval of 0.97–0.99. The analysis results showed that the sensitivity and specificity of the model at the 75% threshold (90%/98%) and 95% threshold (100%/83%) were also quite high. Additionally, the analysis results of the model showed that at the 75% outbreak threshold, the prediction accuracy was 96% (AUC = 0.982), and at the 95% outbreak threshold, the prediction accuracy was 84% (AUC = 0.984). Some previous studies also utilized GTI search volume and successfully built forecasting models for disease outbreaks in Indonesia, Singapore, Brazil, and China (Althouse et al., 2011; Gluskin et al., 2014; Husnayain et al., 2020; Li et al., 2017; Liu et al., 2016; Romero-Alvarez et al., 2020; Đăng, 2019). Among them, the results of this study were quite similar to those of a study in Singapore by Althouse, with a correlation coefficient of 0.931 and an area under the ROC curve of 0.906 (Althouse et al., 2011). Additionally, the study by Tran Ngoc Dang yielded results similar to those of this study, with a correlation coefficient of 0.92 and an area under the ROC curve of 0.97 (Đăng, 2019).

However, the research findings of Tran Ngoc Dang showed that the best model for forecasting DF cases in Ho Chi Minh City, Vietnam is the Basis TSR model built on a 1-week lag time of GTI search volume, combined with controlling autocorrelation using the logarithmic function of DF cases (i.e., Basis TSR + AC: Lag(log(SXH + 1), 1)) (Đăng, 2019). In contrast, the results of this study indicate that the linear regression model between DF cases and GTI search volume within the same week, removing the autocorrelation of DF cases by the 1-week lag time of logarithm of DF cases add 1 (TSR Lag(GTI,0) + AC: Lag(log(SXH+1),1)) is the best forecasting model for DF cases in BR-VT province. This difference may be due to variations in the study conducted in different regions, population densities, Internet penetration, and frequency of Internet-connected device usage.

Alongside the achieved results, the study also has several limitations. Firstly, due to the small scale, the GTI search volume data mainly focuses on the keyword "sốt xuất huyết" (dengue fever). However, this has opened up a new direction in forecasting research, using digital epidemiological data in BR-VT province. Secondly, the data on DF cases only reflect information from reports by healthcare agencies, potentially missing cases that do not seek treatment at healthcare facilities. Thirdly, our model is only a theoretical statistical model and needs to be combined with real-time data sources from online search websites to create a model using real-time data. Lastly, the model cannot be applied to emerging infectious diseases due to the strong influence of the media in Vietnam, resulting in unreliable search volume data.

### 4.3 Generalizability of the model and its applicability to other regions

Although this study was conducted in BRVT province, the approach of using Google Trends to forecast dengue outbreaks can be adjusted and applied to other regions. However, the generalizability of the model needs to be validated in provinces or cities with different levels of internet penetration. A study conducted in Indonesia found that Google Trends data exhibited strong correlations with dengue incidence and could serve as an early warning tool for outbreaks (Syamsuddin et al., 2020b). Similarly, research in Vietnam analyzing long-term dengue incidence trends highlighted the importance of integrating digital surveillance methods with traditional epidemiological data for more effective outbreak prediction (Gibb et al., 2023). However, areas with lower internet usage rates or less prevalent health-related search behaviors may affect the model's accuracy. For instance, in rural regions of Vietnam, only 30% of the population actively searches for health information online (Nguyen et al., 2023), which may limit the effectiveness of Google Trends in these settings. Therefore, future studies should expand the geographical scope and compare results across regions with varying levels of urbanization, climatic conditions, and internet accessibility. Incorporating climate variables such as temperature, rainfall, and humidity can significantly improve the accuracy of dengue forecasting. A study in southern Thailand has identified factors such as temperature, rainfall, cloud cover, and sea level pressure as important predictors of dengue incidence. Specifically, cloud cover was identified as the most influential factor, three times more influential than temperature and rainfall (Abdulsalam et al., 2022). Similarly, incorporating human mobility data from mobile phones into forecasting models has shown significant improvements in capturing the spatial spread of dengue. A study in Pakistan showed that mobility estimates based on mobile phone data can predict the geographic and temporal spread of outbreaks, both in established and emerging areas (Wesolowski et al., 2015). These findings highlight the importance of multi-source data integration in strengthening disease forecasting models.

### 4.4 Influence of media coverage and public awareness on Google trends data

A significant limitation of Google Trends data is its susceptibility to media campaigns and public awareness, which can introduce biases in the analysis. Periods of intensive dengue prevention campaigns may lead to increased search volumes on Google, even when actual case numbers do not surge. For instance, during the COVID-19 pandemic, public interest in preventive measures spiked globally, influenced by extensive media coverage and public health campaigns, leading to increased online searches regardless of actual case numbers (Ito, 2022). Previous research has also highlighted that external factors, such as news coverage in mass media, can alter internet search behavior, making Google Trends data not entirely representative of real epidemiological situations. Furthermore, the impact of media can vary by region; in Vietnam, government-led public health campaigns have significantly influenced search behavior, as seen during COVID-19, where official announcements led to a surge in online information seeking among the public (Le et al., 2020). To mitigate this limitation, future research should explore integrating multiple data sources, such as social media trends (Facebook, Twitter), queries from health websites, and real-time syndromic surveillance data. This multi-source data approach could enhance the reliability and robustness of the forecasting model, making it more suitable for real-world epidemiological applications.

## 5 Conclusion

In summary, the study demonstrates a strong positive correlation between DF cases and GTI search volume, with a correlation coefficient of 0.94 (0.93–0.96) and a p-value <0.001, indicating a significant relationship between the two variables. The most suitable model selected for early forecasting of DF outbreak risk in the locality is the TSR Lag(GTI,0) + AC: Lag(log(SXH+1),1) model, with a correlation coefficient of 0.98 (0.97–0.99), p-value <0.001, dispersion index of 6.8, AUC (at 75% threshold) of 0.982, and AUC (at 95% threshold) of 0.984, indicating a very good predictive ability of the model. Therefore, applying this model in Vietnam, especially in BRVT province, could enhance the effectiveness of disease prevention measures and protect public health.

## CRediT authorship contribution statement

**Dang Anh Tuan:** Writing – review & editing, Writing – original draft, Visualization, Validation, Supervision, Software, Resources, Project administration, Methodology, Investigation, Funding acquisition, Formal analysis, Data curation, Conceptualization. **Pham Vu Nhat Uyen:** Writing – original draft, Visualization, Validation, Methodology, Investigation, Data curation, Conceptualization.

## Compliance with ethical standards

This article does not involve any studies conducted by the authors that included human participants.

## Funding

This research received no external funding.

## Declaration of competing interest

The authors declare that they have no known competing financial interests or personal relationships that could have appeared to influence the work reported in this paper.
