# Corrigendum to “Early prediction of the outbreak risk of dengue fever in Ba Ria-Vung Tau province, Vietnam: An analysis based on Google trends and statistical models” [Infectious Disease Modelling 10, (3), (September 2025), Pages 743-757]

**DOI:** 10.1016/j.idm.2026.04.012

**Published:** 2026-04-27

**Authors:** Dang Anh Tuan, Pham Vu Nhat Uyen

**Affiliations:** a Faculty of Information Technology, Industrial University of Ho Chi Minh City, Ho Chi Minh City, 70000, Viet Nam; b Faculty of Chemical Engineering, Industrial University of Ho Chi Minh City, Ho Chi Minh City, 70000, Viet Nam

The authors regret that the Acknowledgements section was inadvertently omitted during the final stages of manuscript preparation and proofreading. We believe it is important to recognize the contributions of individuals and institutions that were instrumental to the completion of this research.

The text of Acknowledgement is as follows.

The authors would like to apologise for any inconvenience caused.

